# Spending Time in Nature Serves as a Protective Factor against Problematic Alcohol Use: A Structural Equation Modeling Approach

**DOI:** 10.3390/ijerph192013356

**Published:** 2022-10-16

**Authors:** Shahar Almog, Nichole M. Scaglione, JeeWon Cheong, Jillian M. Rung, Andrea Vásquez Ferreiro, Meredith S. Berry

**Affiliations:** 1 Department of Health Education and Behavior, University of Florida, Gainesville, FL 32611, USA; 2 Department of Psychology, University of Florida, Gainesville, FL 32611, USA

**Keywords:** greenspace, nature exposure, alcohol use, substance use, affect, delay discounting

## Abstract

Alcohol use in the U.S. continues to be a prevalent behavior with the potential for far-reaching personal and public health consequences. Risk factors for problematic drinking include negative affect and impulsive decision-making. Research suggests exposure to nature reduces negative affect, increases positive affect, and reduces impulsive choice. The purpose of the current study was to explore the relationships between exposure to nature (actively going out to nature and the level of greenness around the participant’s daily life), affect, impulsive decision-making, and alcohol use, using structural equation modeling. Cross-sectional data (*N* = 340) collected online on Amazon MTurk were used to test the hypothesized relationships separately for alcohol consumption and alcohol-related problems. Actively spending time in nature was associated with lower negative affect and higher positive affect, while passive exposure to nature was only associated with higher positive affect. In turn, negative affect was positively related to both alcohol measures, while positive affect was related to increased alcohol consumption, but not alcohol-related problems. Impulsive decision-making was not related to nature or alcohol measures. Findings suggest that intentionally spending time in nature may protect against problematic alcohol use by reducing negative affect. These results warrant further research on nature as an adjunct treatment for reducing alcohol and substance-related harms and carry implications for public education and increasing accessibility to natural spaces.

## 1. Introduction

A growing body of literature suggests beneficial effects of exposure to natural environments on health and wellbeing. Exposure to natural spaces is associated with lower mortality rates [1], improved physical health outcomes [2], and improved mental health, including anxiety and depression [3,4]. Experimentally, even a short exposure to natural environments, either in person or on a computer screen, improved affect (i.e., emotional experience or state [5]), reduced stress [6], and restored attention [7]. Nature exposure may also be related to healthier decision-making [8]. Due to the different mechanisms underlying these beneficial effects (e.g., reduced negative affect and improving anxiety and depression, which are frequent co-morbid conditions with substance use [9]), nature exposure was recently discussed as a potential adjunctive treatment for substance use disorders [10]. Although promising, research on the effect of exposure to nature on substance use outcomes is still sparse [11].

A small but growing body of research suggests nature exposure may have protective effects related to substance use. Martin et al. [12] found that greater access to yards and residential views of nature was associated with reduced craving for different substances, including alcohol and nicotine, and those effects were mediated by reduced negative affect. Mapping research using residential greenspace measures, captured by vegetation indices computed from satellite or street level images, suggests beneficial effects on substance use outcomes. For instance, residential nature moderated the relationship between healthy peer context (i.e., assessment of friends’ risky and prosocial behaviors) and substance use. Healthy peer context was associated with reduced substance use among adolescents who lived in more versus less green environments, suggesting that natural contexts facilitate the effects of positive social influence on substance use [13]. In a different study with youth and young adults, residential greenness was associated with reduced binge drinking frequency and reduced odds of nicotine use, but not with alcohol use frequency [14]. These data suggest that nature exposure may have protective effects in the context of substance use, and such protective effects may be particularly applicable for substances that are widely available with frequent use across populations and regions (e.g., alcohol). Nevertheless, researchers have noted that neighborhood greenness may not reflect the level of actual contact with nature that the individual experiences [11], calling for more research discerning between the two.

Alcohol is one of the most commonly used substances globally, with potential for misuse. Harmful alcohol use continues to be a health problem with far reaching consequences on both individual and societal levels. Harmful alcohol use is related to increased mortality, injuries, diseases, mental health conditions, violence, and crime [15,16] and can manifest differently across different age groups [17]. In 2019, nearly 15 million people ages 12 and older had an alcohol use disorder in the United States alone [18]. The widespread use of alcohol highlights the need for examining novel and protective interventions with potential for widespread accessibility. As suggested by Berry et al., nature exposure shows promising potential, but more research is warranted to understand the underlying mechanisms for health programs to target [10].

Two factors addressed in both alcohol research and nature research are affect and impulsive decision-making. Affect is commonly viewed as related to motives to drink. Although drinking to enhance positive emotions is strongly linked to greater alcohol consumption, drinking to cope with negative emotions is widely accepted as a risk factor for developing alcohol-related problems and alcohol use disorder [19,20,21]. Some ecological momentary assessment research, in which data are collected several times a day from an individual’s daily routine, has suggested a temporal link between experiencing negative affect and subsequent alcohol use and alcohol-related problems, whereas positive affect may be linked to increased alcohol use that is not necessarily problematic [22,23]. Relatedly, nature research shows that exposure to natural environments (either images or real nature) was found to reduce negative affect and increase positive affect (for a meta-analysis, see [5]).

Impulsive decision-making, as measured by delay discounting, is another construct associated with alcohol use and influenced by nature exposure. Delay discounting is defined as the tendency to choose a smaller-immediate reward over a larger-delayed reward. Delay discounting is considered a facet of impulsive decision-making [24] and has been associated with maladaptive health behaviors [25,26]. Heavy drinkers or people diagnosed with alcohol use disorder tend to show greater (rapid) delay discounting (more “impulsive” decision-making) compared to healthy controls [27,28]; however, delay discounting was not necessarily associated with alcohol consumption even among individuals with alcohol use disorder [28]. Reduced delay discounting (less “impulsive” decision-making) has been associated with nature accessibility, measured by the level of access to green parks and time spent outdoors [29]. Experimentally, exposure to images of natural environments compared to built environments has been shown to reduce delay discounting [30,31,32]. Taken together, nature exposure may influence mechanisms associated with alcohol use, in particular affect and impulsive decision-making. However, more research is needed to understand whether exposure to nature is associated with behavioral outcomes of alcohol use and to determine what type, dosage, or interactive levels of nature is required for beneficial effects. 

Based on the known relationships between nature and affect and delay discounting, which also play a role in alcohol use, the purpose of the current study was to test the relationships among these variables together, using structural equation modeling (SEM) while differentiating between two aspects of experience with nature. SEM is useful to test complex models, as it allows for simultaneous examination of multiple associations (i.e., regression paths), each accounting for the effects of the other pathways. Thus, in addition to testing affect and delay discounting as mediating mechanisms, two independent variables (i.e., predictors in the model) of experience with nature were included: (1) passive exposure to nature, which assessed the daily level of the nature around the participant’s residence and work environment, and (2) active exposure to nature, which assessed the amount of time the participant purposely and actively spends in a natural environment. With the purpose of informing both prevention and treatment programs, two different models were tested. The outcome of the first model was alcohol consumption, which includes levels of consumption that may or may not be considered harmful (i.e., frequency of use, quantity of use, and frequency of binge drinking). The second model examined alcohol-related problems (e.g., alcohol-related memory loss, feelings of guilt, failing to do what is normally expected, injury). Based on the two bodies of literature (i.e., nature, and alcohol), and accounting for each of the other factors in the model, we hypothesized that:

**Hypothesis** **1:** *Exposure to nature would be negatively associated with negative affect, which in turn would be positively associated with alcohol consumption and with alcohol-related problems.*

**Hypothesis** **2:** *Exposure to nature would be positively associated with positive affect, which in turn would be positively associated with alcohol consumption but not with alcohol-related problems.*

**Hypothesis** **3:** *Exposure to nature would be positively associated with delay discounting area-under-the curve (AUC) scores (i.e., less “impulsive” decision making), which in turn would be negatively associated with alcohol-related problems but not with alcohol consumption.*

Because of the paucity of research on examining passive and active exposure to nature separately, we did not differentially hypothesize the specific nature variables.

## 2. Materials and Methods

### 2.1. Recruitment and Procedures

The current study was conducted online, recruiting participants on the Amazon Mechanical Turk (MTurk) platform from March to October 2021. The survey was posted as a task described as a survey on personal experience, personal preferences, and personality questionnaires. Based on the MTurk features, the survey was visible only to participants who (a) were 18 years of age or older, (b) reported residing in the United States, (c) had at least 500 approved tasks on the platform, and (d) had an approval rate of at least 95% of their previous tasks. Interested individuals clicked on the task to access the Qualtrics survey and were presented with an informed consent document for the screening procedures. Participants gave their consent by clicking that they agreed to participate and were asked to complete several (unpaid) screening questions to ensure English proficiency. If adequate American English was demonstrated, the participants continued to the main survey, where they reviewed an informed consent document for the remainder of the study procedures and were asked if they agreed to participate.

In the remaining survey, participants were first presented with the delay discounting task, followed by scales and demographics (each measure described in detail below). In addition to the measures described hereafter, the survey included questions on subjective health and wellbeing, habits, health behaviors, and other substance use. Following recommendations for best practices conducted on MTurk [33], to ensure good quality data, several quality measures were included before arriving at the final sample (described in detail below). The median duration of the survey (with the screener) was 25 min, and the compensation was USD 3.00 (i.e., USD 7.20 hourly pay). At the end of the survey, participants were provided with a completion code to paste back into MTurk. All participants who submitted a valid completion code received compensation. All study procedures were approved by the Institutional Review Board of the University of Florida under IRB202100096 and IRB202201088.

**Figure 1:**
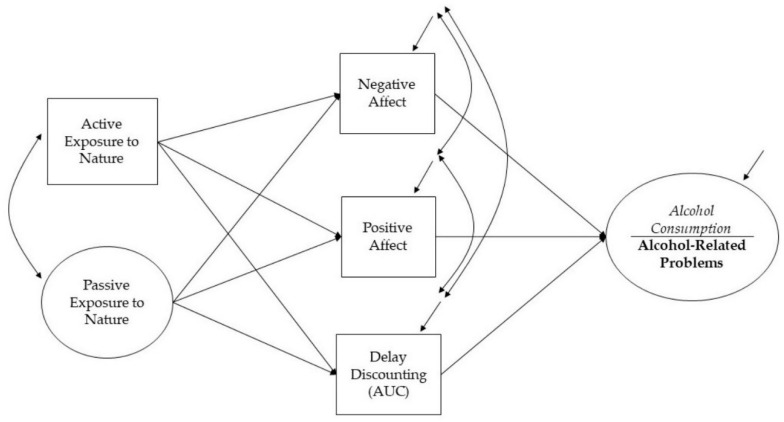
The hypothesized models. Alcohol consumption (model 1) and alcohol-related problems (model 2) regressed on negative affect, positive affect, and delay discounting, which in turn regressed on active exposure to nature and passive exposure to nature.

The measures are presented from left to right on the hypothesized model (See Figure 1).

### 2.2. Active Exposure to Nature 

To assess active exposure to nature, participants were asked for the frequency and duration of visits to natural spaces (based on [34]). Frequency of visits was reported on a categorical scale from 1 (never) to 9 (6–7 days per week); The duration of the visits was reported on a scale ranging from 1 (30 min or less) to 6 (more than 4 h). The sum of the two items was used as an observed, or directly measured, variable (commonly depicted as a rectangle, see Figure 1), where higher values indicate greater active exposure to nature.

### 2.3. Passive Exposure to Nature 

To assess passive exposure to nature, participants responded to six items (based on [29]) that captured the level of nature surrounding their home and work environment (e.g., “From the main living space in your home, is the view outside the window(s) mostly of concrete, buildings, etc. or mostly of natural elements like trees, grass, waterways, etc.?”). Responses were recorded on a scale of 1 (mostly concrete) to 7 (mostly greenspace). The six items were used as indicators of the latent factor (i.e., an unmeasured variable estimated based on participant responses to several measured items, commonly depicted as a circle; see Figure 1) named Passive Exposure to Nature. Higher scores indicated greater passive exposure to nature. Cronbach’s alpha was 0.88.

### 2.4. Affect

To assess positive and negative affect, participants completed the brief version of the Positive and Negative Affect Schedule (PANAS-GEN, [35]). Participants were asked to indicate the extent of their positive and negative feelings during the past week from 1 (very slightly or not at all) to 5 (extremely). The participants responded on ten positive feelings (e.g., interested, inspired) and ten negative feelings (e.g., distressed, ashamed). The positive affect score is the sum of the ten positive items, and the negative affect score is the sum of the ten negative items. Higher scores indicate more of the affect. Cronbach’s alpha was 0.94 for both the positive and the negative affect scales independently. The sum scores were used as observed variables in the hypothesized SEM.

### 2.5. Delay Discounting 

As a measure of impulsive choice, we used a hypothetical titrating amount delay discounting task [36,37]. Participants were presented with a series of questions and were asked to choose between two different monetary rewards; a smaller reward received immediately or a larger reward received after a delay (e.g., “Would you rather have $50 now or $100 in one week?”). In the current task, we used seven delays (1 week, 2 weeks, 1 month, 6 months, 1 year, 5 years, 25 years) with six trials in each delay block. The larger-later reward was always $100, and the first smaller-sooner reward in a block was $50, which changed according to the participant’s choice. If the larger-later reward was chosen, the next smaller-sooner reward increased. If the smaller-sooner reward was chosen, the next smaller-sooner reward decreased. The amount of the smaller-sooner reward was adjusted and reduced by half from trial to trial, starting with $25 after the first choice, then $12.50 in the second, $6.25 in the third, etc. The value of the immediate titrated outcome resulting from the final choice within each delay block was registered as the indifference point or the value at which the smaller sooner and larger later outcomes are subjectively equal. When these seven indifference points are plotted, the areas of the trapezoids under the curve are summed and divided by the total graphical area to provide an area under the curve (AUC) score [38]. The AUC scores can range from 0 to 1, where lower scores mean greater delay discounting (more “impulsive” decision-making), and higher scores mean less discounting (less “impulsive” decision-making). 

### 2.6. Alcohol Use

To assess both alcohol consumption and alcohol-related problems, participants completed the Alcohol Use Disorders Identification Test (AUDIT; [39,40]). The AUDIT includes 10 items that assess patterns of alcohol use, misuse, and related problems. Usually, the sum score of the 10 items is calculated. A score of 8 to 12 indicates harmful drinking, 13 and higher indicates dependent drinking for females, or 15 and higher for males. For the current study, we specified the latent variable of Alcohol Consumption with the first three items (Cronbach’s alpha = 0.80) assessing frequency of drinking, quantity of alcohol consumed on a typical drinking day, and frequency of occasions of heavy drinking (i.e., 6 or more drinks on one occasion). Higher scores indicate greater alcohol consumption. We used the remaining seven items (items 4–10) as indicators of the latent factor Alcohol-Related Problems (Cronbach’s alpha = 0.89). These items capture alcohol-related consequences such as failing to do what is normally expected, injuries, loss of memory, feeling regret, etc. Higher scores reflect a higher frequency of alcohol-related problems.

### 2.7. Data Quality Checks and Cleaning

Following recommendations for best practices on MTurk to ensure good quality data [33], we included several quality measures. Two attention checks were embedded in the delay discounting task, where participants were asked to choose between two rewards (smaller sooner and larger later rewards). In the attention checks, one sensible choice is obvious (e.g., “Would you prefer $0 now or $100 in two weeks?”) and failing to choose it was considered as failing the attention check. Two other instructional attention checks were embedded in other parts of the survey. One item asked participants to remember a word for a later question, and the second item instructed them to choose the “Other” response rather than answering the question truthfully. Failing to remember the specific word or not choosing “Other” was considered failing the attention check. Lastly, two consistency checks, for which a similar question was asked in the screener and the main survey (e.g., year of birth and age) were included. Failing to provide a consistent response on both matching items was considered as failing the quality check. Based on recommendation to treat inattention as a continuum rather than determining quality based on a single marker [41], and the questionable validity of the attention checks embedded in the delay discounting task [42], participant data were excluded from analysis only if they failed two or more quality checks.

Towards the end of the survey, participants were asked whether we, the researchers, should use their data. Participants who explicitly responded not to use their data were excluded from the analysis. Additionally, we used Qualtrics features that flag suspicious responses as bots to be considered for exclusion if they also failed an attention check or provided unreasonable responding patterns (e.g., all scale responses were “1”). 

As a standard practice, to prepare the delay discounting data for analysis, we followed the Johnson and Bickel algorithms to identify nonsystematic data [43]. The first criterion flags data as nonsystematic if there is an increase of 20% or more of the larger-later reward (i.e., $20 in our task) in any indifference point relative to the preceding one. The second criterion flags data as nonsystematic if the reduction from the first indifference point to the last one is smaller than 10% of the larger later reward (i.e., $10 or less in our task). Nonsystematic data may result from misunderstanding the task instructions or inattentive responses. Beyond the traditional utility of the criteria for conceptualizing discounting (i.e., a reduction in value due to a delay) and to enable analysis, nonsystematic discounting data were recently discussed as an indicator of poor quality data in MTurk samples [44]; thus, nonsystematic delay discounting data were excluded from the analysis listwise.

From 2196 recorded entries to the screener, 450 participants completed the main survey. After excluding 110 observations (24.4%), the final analysis sample included 340 participants with no missing data. Data observations were excluded due to multiple attempts to pass the screener by modifying responses (*n* = 19), responding twice without modifying responses over a two-week period; hence, the later observation was excluded (*n* = 1), being flagged by Qualtrics as suspicious accounts (*n* = 13), explicit comments not to use the data (*n* = 3), failing two or more attention and consistency checks (*n* = 32), providing nonsystematic delay discounting data (*n* = 33), and missing exogenous demographic data (*n* = 9). 

### 2.8. Analytic Plan

Descriptive statistics of the analyzed sample were obtained using SPSS 28, including means and standard deviations (or median and first and third quartiles when appropriate) and correlations among the measures. All variables were inspected for normality. The structural equation models were estimated using MPlus (version 8.4) [45]. To account for non-normally distributed items of the AUDIT, the models were estimated with the robust maximum likelihood (MLR) estimator. Overall model fit was evaluated with several global fit indices [46], including Chi-square, root mean square error of approximation (RMSEA), comparative fit index (CFI, [47]), and the standardized root mean squared residual (SRMR). 

Three latent variables were specified: Passive nature exposure with six indicators, alcohol consumption with three indicators, and alcohol-related problems with seven indicators. First, to ensure the indicators were appropriate measures of the relevant latent constructs, we tested measurement models separately for the two alcohol outcomes, where all the study variables were correlated. We evaluated the overall model fit and the significance and magnitude of each indicator. 

Next, we examined two structural equation models where we regressed alcohol consumption (model 1) or alcohol-related problems (model 2) onto negative affect, positive affect, and delay discounting, which were regressed onto active and passive exposure to nature. Age, gender, and income were included as covariates related to all endogenous variables (i.e., negative affect, positive affect, delay discounting, and alcohol use outcomes). As the active and passive exposures to nature variables were expected to be correlated, the two exogenous variables were specified to covary. The residual variances of the negative and positive affect and delay discounting were also correlated. Lastly, although the data were cross-sectional, we utilized mediation analysis to explore the potential mediational pathways from exposure to nature to alcohol outcomes via positive affect, negative affect, and delay discounting. Mediated effects were estimated and tested using the product of the coefficient method embedded in MPlus [48,49]. 

## 3. Results

### 3.1. Participants 

The participants in the final sample (*N* = 340) were an average of 40.9 years old (SD = 12.50, range 19–77), 50.9% identified as female, and 80.3% identified as white (sample demographics are presented in Table 1). Approximately one-third of the participants (33.5%) reported never drinking alcohol, resulting in 66.5% who reported any alcohol use. This percentage generally aligns with the U.S. population, where 69.5% of adults reported drinking alcohol in the past year [18]. Based on the standard scoring of the AUDIT, 8.2% of the current sample reported harmful alcohol drinking, and 6.8% reported dependent drinking. Overall, this sample represents the general population with different levels of alcohol use, rather than an explicit focus on individuals with alcohol use disorder.

### 3.2. Model Fit

Correlations, means, and standard deviations of all measures are presented in the supplemental Table S1. The hypothesized measurement models fit the data at an acceptable to good level with RMSEAs < 0.068, CFIs > 0.928, and SRMRs < 0.069 (see supplemental Table S2). Loadings and significance of the indicators are presented in Table 2. All indicators of the three latent factors (i.e., passive nature exposure, alcohol consumption, alcohol-related problems) were significant (*p* < 0.01). All indicators but one had loadings higher than 0.4, suggesting the indicators were related to the latent factor. Item 9 of the AUDIT (“Have you or someone else been injured as a result of your drinking?”) in model 2 had a lower loading on the latent factor Alcohol-Related Problems (standardized estimate = 0.234, *p* = 0.009). However, since the AUDIT is a well-established questionnaire, Cronbach’s alpha showed good internal consistency of 0.89, and the specific item captures a more rare and severe consequence of alcohol-related problems, the item was retained. Taken together, the measurement models were satisfactory and allowed us to proceed with the structural models.

### 3.3. Hypothesis Testing

**Figure 2:**
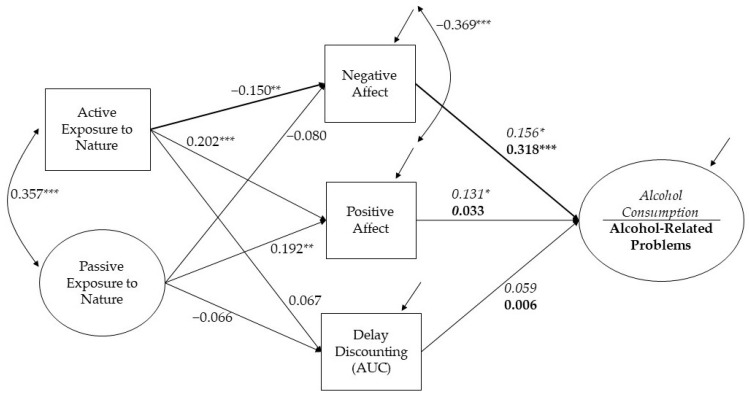
The relationships among nature, affect, delay discounting, and alcohol, controlling for age, gender, and income. All coefficients are standardized estimates with * *p* < 0.05; ** *p* < 0.01; *** *p* < 0.001. The estimates in regular font are identical in both models, the italic font is unique for Model 1 (Alcohol Consumption), and the bolded font is unique for Model 2 (Alcohol-Related Problems). Bolded arrows represent the significant mediated effect of active exposure to nature on alcohol-related problems. Nonsignificant correlations among the residuals of positive affect, negative affect, and delay discounting were not reported for simplicity.

After nonsignificant paths of the covariates (i.e., age, gender, income) were set to zero and residuals of potential mediators were correlated, the structural models fit the data at acceptable to good levels (see supplemental Table S2). For model 1 (Alcohol Consumption) fit indices were, χ^2^(90) = 218.760 (*p* < 0.001), RMSEA = 0.065, CFI = 0.928 and SRMR = 0.073. Fit indices for model 2 (Alcohol-Related Problems) were, χ^2^(157) = 259.900 (*p* < 0.001), RMSEA = 0.044, CFI = 0.948 and SRMR = 0.064. Figure 2 shows the standardized estimates of both models.

Supporting our hypothesis, active exposure to nature was negatively associated with negative affect (β = −0.150, SE = 0.055, *p* = 0.006), which in turn was positively associated with alcohol consumption (β = 0.156, SE = 0.062, *p* = 0.012) and alcohol-related problems (β = 0.318, SE = 0.073, *p* < 0.001). The product of coefficients revealed a significant indirect effect of active exposure to nature on alcohol-related problems through negative affect (β = −0.048, SE = 0.022, *p* = 0.028), suggesting active exposure to nature may be a protective factor against problematic alcohol consequences by reducing one’s negative affect. The other indirect effects via negative affect on alcohol consumption did not reach conventional levels of significance (β = −0.023, SE = 0.013, *p* = 0.075).

As hypothesized, both active and passive exposure to nature were positively associated with positive affect (standardized coefficients of active exposure to nature β = 0.202, SE = 0.057, *p* < 0.001, and passive exposure to nature β = 0.192, SE = 0.061, *p* = 0.002), which in turn was positively associated with alcohol consumption (β = 0.131, SE = 0.062, *p* = 0.036) but not with alcohol-related problems (β = 0.033, SE = 0.064, *p* = 0.610). Despite significant relevant paths, indirect effects via positive affect on alcohol consumption did not reach conventional levels of significance for active or passive exposure to nature (β = 0.026, SE = 0.015, *p* = 0.077 and β = 0.025, SE = 0.015, *p* = 0.087, respectively). 

In contrast to our hypothesis, delay discounting AUC was not significantly associated with either of the nature variables (*p*s > 0.207) or alcohol-related problems (*p* = 0.928).

As an exploratory secondary analysis, we excluded participants who reported no alcohol use and analyzed the sub-sample of people reporting some alcohol use (*n* = 226). The models yielded similar results in which the indirect effect of active exposure to nature on reduced alcohol-related problems through reduced negative affect remained consistent (β = −0.075, SE = 0.033, *p* = 0.025).

### 3.4. Covariates and Residuals

Regarding the covariates, being younger and female were associated with greater delay discounting, that is, more “impulsive” decision-making, (β = 0.159, SE = 0.053, *p* = 0.003 and β = −0.127, SE = 0.055, *p* = 0.021, respectively) and greater negative affect (β = −0.213, SE = 0.044, *p* < 0.001 and β = 0.169, SE = 0.048, *p* < 0.001, respectively). Younger age was also associated with greater alcohol consumption (β = −0.168, SE = 0.049, *p* = 0.001). Income was not associated with any of the endogenous variables. Passive and active exposure to nature were positively correlated (r = 0.357, *p* < 0.001), and, as expected, residuals of positive affect and negative affect were negatively correlated (r = −0.369, *p* < 0.001).

## 4. Discussion

The purpose of this study was to examine the relationships between exposure to nature and alcohol use through the underlying factors of affect and delay discounting. Because passive exposure to nature (i.e., the daily level of greenness around the participant’s home and work environment) may not reflect the individual’s deliberate experience and time spent in natural spaces, we assessed both passive and active aspects of exposure to nature. We assessed two alcohol outcomes—alcohol consumption (model 1) and alcohol-related problems (model 2), with three mediators—negative affect, positive affect, and delay discounting. 

Spending time in nature (i.e., active exposure) was associated with reduced negative affect, which in turn, and consistent with the alcohol literature [22], was positively associated with both alcohol consumption (model 1) and alcohol-related problems (model 2). Taken together, intentionally spending time in nature may reduce problematic alcohol use (i.e., increased consumption and harmful consequences) by reducing one’s negative affect. This finding supports Berry et al.’s [10] suggestion that nature exposure may have beneficial effects that make it apt to serve as an adjunctive treatment to substance use disorders (and in this case, alcohol), although more research is needed to understand causal influences of nature on substance use.

Focusing on the positive affect paths, results showed that both aspects of nature exposure (i.e., active and passive) were associated with increased positive affect, which in turn was associated with increased alcohol consumption (model 1), but not with alcohol-related problems (model 2). These results suggest that although nature exposure may be related to increased alcohol consumption, consumption that is related to positive emotions may not necessarily be problematic. Moreover, common co-morbid conditions to substance use include affective-related conditions (e.g., anxiety, depression, stress) [9]; thus, our findings that nature was associated with improved affect overall (both higher positive affect and lower negative affect) appear to suggest an additional indirect benefit on substance use.

The structural equation models successfully distinguished between different processes. First, the models were able to distinguish between alcohol consumption that was related to negative affect, which was also related to harmful consequences, and alcohol consumption that was related to positive affect and may not be as problematic. Second, the models provided supportive evidence that residential greenness may not have the same impact as direct contact with nature and that this distinction is necessary to better understand nature’s potential protective effects. While in our study the models revealed a positive association between passive exposure to nature and alcohol consumption (indirect effect at trend level, mediated by positive affect), Wiley et al. [14] found a negative association between nature exposure and binge drinking frequency and no association with alcohol use frequency. Wiley et al., however, only included a measure of residential greenness (similar to our passive exposure). Our findings revealed that actively going out to nature was associated with reduced problematic use of alcohol (both consumption and related problems), which appears to function, in part, through reduced negative affect. Our study found a moderate correlation between passive and active exposure to nature, which may suggest that some people who are exposed to nature are also more likely to be engaged with it. As such, it is possible that Wiley et al.’s negative association between passive nature exposure and binge drinking was confounded by the beneficial effect of actively spending time in natural spaces, which was not measured in that study [14]. Still, it is possible that nature effects binge drinking behavior differently than frequency of drinking alcohol, which can include a wider range of drinking behaviors when tested separately. Additionally, our sample was older than Wiley et al.’s sample (mean age of ~41 vs. ~20, respectively). Because binge drinking is more common among younger adults [17], future research would benefit from investigating whether the relationship between nature exposure and alcohol use is moderated by age.

Contrary to our hypotheses, the delay discounting paths were nonsignificant. Repke et al. [29] found that nature accessibility was related to reduced delay discounting (i.e., reduced impulsivity), and there were beneficial indirect effects (through reduced delay discounting) on depression, anxiety, and stress, as well as on general health and wellbeing. Unlike Repke et al.’s findings, our study did not reveal any relationship between nature and delay discounting. Our models also did not reveal any significant association between delay discounting and alcohol-related problems. In the alcohol literature, greater delay discounting is associated with alcohol use disorder [27]; however, our sample was a general sample, with only 15% categorized as engaging in harmful drinking according to the AUDIT scores, which may limit direct comparisons. In our sample, delay discounting was also not associated with alcohol consumption, which is consistent with Gowin et al. [28], who found that although individuals without a diagnosis of alcohol use disorder discounted less than people with alcohol use disorder, in both populations, delay discounting was not associated with alcohol consumption in the prior 90 days. In summary, our findings suggest that within this general population, the association between nature and alcohol-related problems and alcohol consumption is not significantly explained by delay discounting.

Our findings have several implications for education and policy. Although passive and active exposure to nature were correlated, suggesting that closeness to natural spaces provides more opportunities to connect with nature, one’s proximity to natural environments alone might not contribute to regulating negative emotions. The nature around us might be taken for granted, as one of the participants commented: “This study made me look out the glass door into my backyard and appreciate my pool, pond, grass, and colorful plants.” Taken for granted (e.g., divided attention, passively experienced), nature may not be beneficial in regulating negative emotions that are related to problematic alcohol use. Together, our findings support advocacy to improve access to natural spaces, especially in urban residential areas that are often deprived of closeness to nature. Having access to greenspaces is the first step. Next, since people tend to underestimate the capacity of exposure to nature to improve their mood [50], education on the importance of spending time in nature is necessary. Education is especially important for specific populations prone to developing problematic alcohol use (e.g., college students that engage in binge drinking) as our findings suggest that actively engaging with nature has the potential to reduce harmful consumption and related problems.

Significant covariate paths showed that delay discounting and negative affect were significantly associated with age and gender. Being younger and female was associated with greater impulsive decision-making and greater negative affect. These findings align with past research on age and delay discounting [51], and gender and negative affect [52]. Younger age was also associated with greater alcohol consumption but not alcohol-related problems. Future research may look into age and gender-based differences and whether alcohol use in different populations might be affected differently by nature.

Other than the cross-sectional nature of the data, which prohibits concluding causality, the primary limitations of this study are that the data were self-reported and collected online using the MTurk platform. First, literature on research conducted on MTurk highlights various challenges, from participant misrepresentation to inattentive responding. To address these issues, we used several validity measures and checks (e.g., a screener to verify adequate American English proficiency, quality checks embedded in the survey) and well-established criteria to exclude poor quality data, leaving us confident in the integrity of the data analyzed. Second, beyond quality data issues on MTurk, any self-report data (particularly regarding sensitive topics such as substance use) relies on participants’ honesty and memory. Although there is some evidence that the population of alcohol users on MTurk provides valid and reliable data [53], this limitation remains in the current study.

Relatedly, our study recruited a general sample. Although drinking prevalence was generally comparable in percentages to the U.S. population, it is possible that individuals who struggle with severe alcohol use disorder are less represented on MTurk; thus, our results may not be relevant to that specific population. Future randomized controlled trials might systematically examine the effects of nature exposure on alcohol use in treatment contexts. Specifically, alcohol use and affective states could be measured before and after treatment in conjunction with active nature exposure versus treatment as usual with no active nature exposure or other appropriate control condition. An experimental approach would offer complementary rigor to cross-sectional research in order to isolate the effects of nature exposure as an adjunctive component to traditional forms of substance use treatment.

Moreover, it is possible that the beneficial effects of nature may vary across different sub-groups of individuals who use alcohol. For example, nature exposure may be more likely to yield protective effects for those with moderate compared to higher levels of alcohol consumption. Conversely, there may be greater potential for beneficial effects of nature among heavier users of alcohol. The present study was underpowered to test such hypotheses; however, these questions represent an important area of future research to determine the effects of nature exposure on varying levels of alcohol use and related variables (e.g., affect).

Future research may also benefit from measuring the frequency and duration of nature visits more directly to establish a “dose-response” recommendation, preferably utilizing ecological momentary assessment tools and longitudinal designs that will elucidate any temporal relationships between exposure to natural environments and alcohol use. Similarly, prevention and treatment programs may benefit from including nature exposure elements, from which the adjunctive nature effect on program outcomes can be examined.

## 5. Conclusions

The findings of the current study suggest that actively spending time in nature may be beneficial against problematic alcohol use through reduced negative affect. Spending time in nature could provide an accessible alternative activity to alcohol use with additional beneficial effects on wellbeing and/or serve as an adjunctive tool in prevention and treatment programs for harmful alcohol use. These findings set the stage for future research to explore such effects in clinical populations, differentiate between residential greenness and level of contact with nature, use more accurate and temporal measures of nature exposure related to alcohol outcomes, and examine outcomes of programs that will include nature exposure as an adjunct treatment.

## Figures and Tables

**Table 1:** Demographics of the analyzed sample.

Demographics	*N* = 340
Age, Mean (*SD*)	40.9 (12.50)
Gender, *n* (%)	
Males	167 (49.1%)
Females	173 (50.9%)
Race, *n* (%)	
Asian	29 (8.5%)
Black or African American	18 (5.3%)
White	273 (80.3%)
Mixed	12 (3.5%)
Other	8 (2.4%)
Hispanic/LatinX, *n* (%)	26 (7.6%)
Education, *n* (%)	
High school graduate or less	46 (13.5%)
Some college (no degree)	63 (18.5%)
Associate degree	38 (11.2%)
Bachelor’s degree	142 (41.8)
Master’s degree	42 (12.4%)
Doctoral or Professional degree (JD, MD)	9 (2.6%)
Income, *n* (%)	
USD 29,999 or less	117 (34.4%)
USD 30,000–59,999	121 (35.6%)
USD 60,000–89,999	53 (15.6%)
USD 90,000–119,999	24 (7.0%)
USD 120,000 or more	25 (7.4%)

Note. Income = annual income.

**Table 2:** The standardized estimates, standard errors, and *p*-values of indicators of the latent variables in both models.

		Model 1 Alcohol Consumption			Model 2 Alcohol-Related Problems		
	Indicator Item	Estimate	Standard Error	*p*-Value (Two-Tailed)	Estimate	Standard Error	*p*-Value (Two-Tailed)
Passive Exposure to Nature							
	NP1	0.902	0.016	*p* < 0.001	0.903	0.016	*p* < 0.001
	NP2	0.869	0.025	*p* < 0.001	0.868	0.025	*p* < 0.001
	NP3	0.807	0.023	*p* < 0.001	0.806	0.023	*p* < 0.001
	NP5	0.832	0.026	*p* < 0.001	0.833	0.026	*p* < 0.001
	NP6	0.677	0.036	*p* < 0.001	0.677	0.036	*p* < 0.001
	NP8	0.438	0.051	*p* < 0.001	0.439	0.051	*p* < 0.001
Alcohol Consumption							
	AUDIT Q1	0.602	0.036	*p* < 0.001			
	AUDIT Q2	0.811	0.038	*p* < 0.001			
	AUDIT Q3	0.959	0.026	*p* < 0.001			
Alcohol-Related Problems							
	AUDIT Q4				0.886	0.034	*p* < 0.001
	AUDIT Q5				0.867	0.041	*p* < 0.001
	AUDIT Q6				0.785	0.063	*p* < 0.001
	AUDIT Q7				0.858	0.046	*p* < 0.001
	AUDIT Q8				0.824	0.047	*p* < 0.001
	AUDIT Q9				0.234	0.090	*p* = 0.009
	AUDIT Q10				0.488	0.089	*p* < 0.001

Note. NP = Nature Passive, AUDIT = Alcohol Use Disorders Identification Test, Q = Question.

## Data Availability

The data presented in this study are openly available in OSF at https://osf.io/q5wkh/?view_only=0d92b4c61d224a3ab2fe46a89c41c254.

## Supplementary Materials

The following supporting information can be downloaded at: https://www.mdpi.com/article/10.3390/ijerph192013356/s1, Table S1: Pearson Correlations, Means and Standard Deviations of the Measures.; Table S2: Model Fit Indices.
