# Supplementary material for: Spending Time in Nature Serves as a Protective Factor against Problematic Alcohol Use: A Structural Equation Modeling Approach
Source: Int J Environ Res Public Health. 2022 Oct 16;19(20):13356. doi: 10.3390/ijerph192013356 (PMC9603149; doi:10.3390/ijerph192013356)
Supplement: Supplementary file 1 [file ijerph-19-13356-s001.zip › ijerph-1914973-supplementary.pdf]

Spending Time in Nature Serves as a Protective Factor Against Problematic Alcohol Use: A Structural Equation Modeling Approach

Shahar Almog, Nichole M. Scaglione, JeeWon Cheong, Jillian M. Rung, Andrea Vásquez Ferreiro & Meredith S. Berry

Supplementary Material

Table S1. Pearson correlations, means and standard deviations of the measures.

	Active Nature	Passive Nature						Affect (PANAS)		DD	AUDIT									
	NatAct	NP1	NP2	NP3	NP5	NP6	NP8	Positive PA_score	Negative NA_score	DD	AUDIT 1	AUDIT 2	AUDIT 3	AUDIT 4	AUDIT 5	AUDIT 6	AUDIT 7	AUDIT 8	AUDIT 9	AUDIT 10
NP1	0.335																			
NP2	0.263	0.794																		
NP3	0.234	0.713	0.754																	
NP5	0.357	0.751	0.684	0.656																
NP6	0.283	0.588	0.554	0.515	0.675															
NP8	0.182	0.434	0.367	0.315	0.365	0.241														
PA_score	0.270	0.212	0.206	0.201	0.281	0.227	0.130													
NA_score	-0.207	-0.133	-0.129	-0.103	-0.184	-0.095	-0.109	-0.401												
DD	0.066	-0.004	-0.042	-0.017	-0.016	-0.017	-0.018	-0.076	-0.006											
AUDIT1	0.129	0.058	0.020	-0.027	0.002	0.048	-0.015	0.068	0.058	0.035										
AUDIT2	-0.019	-0.019	-0.052	-0.037	-0.017	0.035	0.038	0.010	0.120	0.035	0.488									
AUDIT3	-0.017	-0.023	-0.064	-0.078	-0.035	0.040	0.006	0.055	0.137	0.020	0.579	0.779								
AUDIT4	-0.097	0.057	-0.011	-0.040	-0.025	0.051	0.059	-0.070	0.269	0.025	0.421	0.551	0.694							
AUDIT5	-0.102	0.035	-0.023	-0.038	-0.025	0.061	0.020	-0.100	0.233	-0.012	0.391	0.484	0.620	0.758						
AUDIT6	-0.027	0.080	0.012	0.022	0.027	0.042	0.028	-0.021	0.228	-0.006	0.334	0.315	0.499	0.721	0.696					
AUDIT7	-0.075	-0.048	-0.083	-0.109	-0.074	0.018	-0.036	-0.131	0.278	0.004	0.411	0.497	0.618	0.744	0.775	0.628				
AUDIT8	-0.043	0.048	-0.017	-0.049	-0.016	0.035	0.020	-0.055	0.250	-0.003	0.428	0.552	0.674	0.736	0.689	0.653	0.722			
AUDIT9	-0.095	-0.046	-0.015	-0.026	-0.044	-0.053	-0.025	-0.038	0.278	-0.012	0.129	0.077	0.198	0.178	0.169	0.210	0.224	0.154		
AUDIT10	-0.101	0.040	.001	0.041	-0.042	0.036	0.098	-0.069	0.243	-0.048	0.282	0.338	0.407	0.453	0.408	0.317	0.422	0.400	0.327	
Mean	8.81	4.81	4.68	4.64	4.55	4.65	3.56	32.05	16.63	0.220	1.42	0.46	0.49	0.21	0.16	0.12	0.24	0.21	0.11	0.30
SD	2.437	1.726	1.845	2.015	1.574	1.261	1.974	9.589	7.827	0.207	1.323	0.870	0.914	0.673	0.561	0.502	0.667	0.637	0.498	0.891

Note. *N* = 340, NatAct = Active exposure to nature, NP = Passive exposure to nature, PA = Positive Affect, NA = Negative Affect, DD = Delay discounting AUC, AUDIT = Alcohol Use Disorders Identification Test, SD = Standard Deviation

Table S2. Fit indices of measurement and structural models.

		$\chi^2(df)$	RMSEA	CFI	SRMR
**Model 1: Alcohol Consumption**					
	Measurement Model	216.972 (85) *p* < .001	0.068	0.928	0.069
	Structural Model	218.760 (90) *p* < .001	0.065	0.928	0.073
**Model 2: Alcohol Related Problems**					
	Measurement Model	257.255 (151) *p* < .001	0.045	0.947	0.064
	Structural Model	259.900 (157) *p* < .001	0.044	0.948	0.064

Note. Measurement models include all covariates paths. In the structural models nonsignificant covariate paths were set to zero.
